# Supplementary material for: Hybridization alters the shape of the genotypic fitness landscape, increasing access to novel fitness peaks during adaptive radiation
Source: eLife. 2022 May 26;11:e72905. doi: 10.7554/eLife.72905 (PMC9135402; doi:10.7554/eLife.72905)
Supplement: Supplementary file 1. — (a)—Table 1. Samples of hybrids and parental studies used either in genomic or in morphological analyses, along with associated metadata. (b)—Table 2. Models tested to assess the extent to which specialist ancestry predicts measures of fitness and their respective fits using all samples and an unsupervised ADMIXTURE analysis. Best-fit models are bolded. (c)—Table 3. Models tested to assess the extent to which specialist ancestry predicts measures of fitness and their respective fits using all samples and a supervised ADMIXTURE analysis. Best-fit models are bolded. (d)—Table 4. Models tested to assess the extent to which specialist ancestry predicts measures of fitness and their respective fits using only samples from the second field experiment (Martin and Gould, 2020) and an unsupervised ADMIXTURE analysis. Best-fit models are bolded. (e)—Table 5. Models tested to assess the extent to which genome-wide variation (PC1/PC2) predicts measures of fitness and their respective fits using all samples and an unsupervised ADMIXTURE analysis. Best-fit models are bolded. (f)—Table 6. Single nucleotide polymorphisms (SNPs) found to be strongly associated with composite fitness using SnpEff (Cingolani et al., 2012). SNPs that were identified as being strongly associated with both growth and composite fitness are italicized, and those that remain significant after a Bonferroni correction are bolded. (g)—Table 7. Gene ontology term enrichment for genes associated with composite fitness. (h)—Table 8. SNPs found to be strongly associated with growth SnpEff (Cingolani et al., 2012). SNPs that were identified as being strongly associated with both growth and composite fitness are italicized, and those that remain significant after a Bonferroni correction are bolded, (i)—Table 9. Gene ontology term enrichment for genes associated with growth. (j)—Table 10. List of the 31 morphological traits measured for this study and standard length; corresponding landmark IDs match those shown in F [file elife-72905-supp1.docx]

**Supplementary file 1**

**Supplementary file 1a**

| ID | Sequenced | Morphology | | Survivorship | Experiment | Lake | Species |
| --- | --- | --- | --- | --- | --- | --- | --- |
| CP04E02 | This study | | Yes | Non-Survivor | Martin & Gould 2020 | Crescent Pond | Hybrid |
| CP07F05 | This study | | Yes | Non-Survivor | Martin & Gould 2020 | Crescent Pond | Hybrid |
| CP08H06 | This study | | Yes | Non-Survivor | Martin & Gould 2020 | Crescent Pond | Hybrid |
| CP09C02 | This study | | Yes | Non-Survivor | Martin & Gould 2020 | Crescent Pond | Hybrid |
| CP09D01 | This study | | Yes | Non-Survivor | Martin & Gould 2020 | Crescent Pond | Hybrid |
| CP09F10 | This study | | Yes | Non-Survivor | Martin & Gould 2020 | Crescent Pond | Hybrid |
| CP10B05 | This study | | Yes | Non-Survivor | Martin & Gould 2020 | Crescent Pond | Hybrid |
| CP11C10 | This study | | Yes | Non-Survivor | Martin & Gould 2020 | Crescent Pond | Hybrid |
| CP13E03 | This study | | Yes | Non-Survivor | Martin & Gould 2020 | Crescent Pond | Hybrid |
| CP13F01 | This study | | Yes | Non-Survivor | Martin & Gould 2020 | Crescent Pond | Hybrid |
| LL01F03 | This study | | Yes | Non-Survivor | Martin & Gould 2020 | Little Lake | Hybrid |
| LL01F05 | This study | | Yes | Non-Survivor | Martin & Gould 2020 | Little Lake | Hybrid |
| LL01G05 | This study | | Yes | Non-Survivor | Martin & Gould 2020 | Little Lake | Hybrid |
| LL02A08 | This study | | Yes | Non-Survivor | Martin & Gould 2020 | Little Lake | Hybrid |
| LL02D09 | This study | | Yes | Non-Survivor | Martin & Gould 2020 | Little Lake | Hybrid |
| LL02D11 | This study | | Yes | Non-Survivor | Martin & Gould 2020 | Little Lake | Hybrid |
| LL02E04 | This study | | Yes | Non-Survivor | Martin & Gould 2020 | Little Lake | Hybrid |
| LL02E07 | This study | | Yes | Non-Survivor | Martin & Gould 2020 | Little Lake | Hybrid |
| LL02E09 | This study | | Yes | Non-Survivor | Martin & Gould 2020 | Little Lake | Hybrid |
| LL04E03 | This study | | Yes | Non-Survivor | Martin & Gould 2020 | Little Lake | Hybrid |
| LL04E09 | This study | | Yes | Non-Survivor | Martin & Gould 2020 | Little Lake | Hybrid |
| LL04F02 | This study | | Yes | Non-Survivor | Martin & Gould 2020 | Little Lake | Hybrid |
| LL04F07 | This study | | Yes | Non-Survivor | Martin & Gould 2020 | Little Lake | Hybrid |
| LL05A01 | This study | | Yes | Non-Survivor | Martin & Gould 2020 | Little Lake | Hybrid |
| LL05C06 | This study | | Yes | Non-Survivor | Martin & Gould 2020 | Little Lake | Hybrid |
| LL05C09 | This study | | Yes | Non-Survivor | Martin & Gould 2020 | Little Lake | Hybrid |
| LL05C10 | This study | | Yes | Non-Survivor | Martin & Gould 2020 | Little Lake | Hybrid |
| LL05D10 | This study | | Yes | Non-Survivor | Martin & Gould 2020 | Little Lake | Hybrid |
| LL05E06 | This study | | Yes | Non-Survivor | Martin & Gould 2020 | Little Lake | Hybrid |
| LL05E08 | This study | | Yes | Non-Survivor | Martin & Gould 2020 | Little Lake | Hybrid |
| LL05H10 | This study | | Yes | Non-Survivor | Martin & Gould 2020 | Little Lake | Hybrid |
| LL06A10 | This study | | Yes | Non-Survivor | Martin & Gould 2020 | Little Lake | Hybrid |
| LL06B03 | This study | | Yes | Non-Survivor | Martin & Gould 2020 | Little Lake | Hybrid |
| LL06C10 | This study | | Yes | Non-Survivor | Martin & Gould 2020 | Little Lake | Hybrid |
| LL06D12 | This study | | Yes | Non-Survivor | Martin & Gould 2020 | Little Lake | Hybrid |
| LL06E10 | This study | | Yes | Non-Survivor | Martin & Gould 2020 | Little Lake | Hybrid |
| LL06F08 | This study | | Yes | Non-Survivor | Martin & Gould 2020 | Little Lake | Hybrid |
| LL08B09 | This study | | Yes | Non-Survivor | Martin & Gould 2020 | Little Lake | Hybrid |
| LL08B11 | This study | | Yes | Non-Survivor | Martin & Gould 2020 | Little Lake | Hybrid |
| LL08G07 | This study | | Yes | Non-Survivor | Martin & Gould 2020 | Little Lake | Hybrid |
| LL08G10 | This study | | Yes | Non-Survivor | Martin & Gould 2020 | Little Lake | Hybrid |
| LL08H01 | This study | | Yes | Non-Survivor | Martin & Gould 2020 | Little Lake | Hybrid |
| LL09A09 | This study | | Yes | Non-Survivor | Martin & Gould 2020 | Little Lake | Hybrid |
| LL09A10 | This study | | Yes | Non-Survivor | Martin & Gould 2020 | Little Lake | Hybrid |
| LL09B05 | This study | | Yes | Non-Survivor | Martin & Gould 2020 | Little Lake | Hybrid |
| LL09C03 | This study | | Yes | Non-Survivor | Martin & Gould 2020 | Little Lake | Hybrid |
| LL09D01 | This study | | Yes | Non-Survivor | Martin & Gould 2020 | Little Lake | Hybrid |
| LL09D10 | This study | | Yes | Non-Survivor | Martin & Gould 2020 | Little Lake | Hybrid |
| LL09E01 | This study | | Yes | Non-Survivor | Martin & Gould 2020 | Little Lake | Hybrid |
| LL09F03 | This study | | Yes | Non-Survivor | Martin & Gould 2020 | Little Lake | Hybrid |
| LL09H07 | This study | | Yes | Non-Survivor | Martin & Gould 2020 | Little Lake | Hybrid |
| LL09H10 | This study | | Yes | Non-Survivor | Martin & Gould 2020 | Little Lake | Hybrid |
| LL10A08 | This study | | Yes | Non-Survivor | Martin & Gould 2020 | Little Lake | Hybrid |
| CP02D01 | This study | | Yes | Survivor | Martin & Gould 2020 | Crescent Pond | Hybrid |
| CP02E09 | This study | | Yes | Survivor | Martin & Gould 2020 | Crescent Pond | Hybrid |
| CP03D02 | This study | | Yes | Survivor | Martin & Gould 2020 | Crescent Pond | Hybrid |
| CP03G03 | This study | | Yes | Survivor | Martin & Gould 2020 | Crescent Pond | Hybrid |
| CP03G07 | This study | | Yes | Survivor | Martin & Gould 2020 | Crescent Pond | Hybrid |
| CP05B08 | This study | | Yes | Survivor | Martin & Gould 2020 | Crescent Pond | Hybrid |
| CP05E02 | This study | | Yes | Survivor | Martin & Gould 2020 | Crescent Pond | Hybrid |
| CP05G12 | This study | | Yes | Survivor | Martin & Gould 2020 | Crescent Pond | Hybrid |
| CP05H10 | This study | | Yes | Survivor | Martin & Gould 2020 | Crescent Pond | Hybrid |
| CP06E09 | This study | | Yes | Survivor | Martin & Gould 2020 | Crescent Pond | Hybrid |
| CP06G01 | This study | | Yes | Survivor | Martin & Gould 2020 | Crescent Pond | Hybrid |
| CP06H09 | This study | | Yes | Survivor | Martin & Gould 2020 | Crescent Pond | Hybrid |
| CP07E08 | This study | | Yes | Survivor | Martin & Gould 2020 | Crescent Pond | Hybrid |
| CP07H01 | This study | | Yes | Survivor | Martin & Gould 2020 | Crescent Pond | Hybrid |
| CP07H11 | This study | | Yes | Survivor | Martin & Gould 2020 | Crescent Pond | Hybrid |
| CP08F07 | This study | | Yes | Survivor | Martin & Gould 2020 | Crescent Pond | Hybrid |
| CP08G08 | This study | | Yes | Survivor | Martin & Gould 2020 | Crescent Pond | Hybrid |
| CP09D02 | This study | | Yes | Survivor | Martin & Gould 2020 | Crescent Pond | Hybrid |
| CP10A03 | This study | | Yes | Survivor | Martin & Gould 2020 | Crescent Pond | Hybrid |
| CP10C04 | This study | | Yes | Survivor | Martin & Gould 2020 | Crescent Pond | Hybrid |
| CP10C07 | This study | | Yes | Survivor | Martin & Gould 2020 | Crescent Pond | Hybrid |
| CP10C12 | This study | | Yes | Survivor | Martin & Gould 2020 | Crescent Pond | Hybrid |
| CP10F12 | This study | | Yes | Survivor | Martin & Gould 2020 | Crescent Pond | Hybrid |
| CP10H01 | This study | | Yes | Survivor | Martin & Gould 2020 | Crescent Pond | Hybrid |
| CP11B03 | This study | | Yes | Survivor | Martin & Gould 2020 | Crescent Pond | Hybrid |
| CP11C08 | This study | | Yes | Survivor | Martin & Gould 2020 | Crescent Pond | Hybrid |
| CP11D04 | This study | | Yes | Survivor | Martin & Gould 2020 | Crescent Pond | Hybrid |
| CP11G06 | This study | | Yes | Survivor | Martin & Gould 2020 | Crescent Pond | Hybrid |
| CP11G12 | This study | | Yes | Survivor | Martin & Gould 2020 | Crescent Pond | Hybrid |
| CP12E05 | This study | | Yes | Survivor | Martin & Gould 2020 | Crescent Pond | Hybrid |
| CP12E07 | This study | | Yes | Survivor | Martin & Gould 2020 | Crescent Pond | Hybrid |
| CP12H04 | This study | | Yes | Survivor | Martin & Gould 2020 | Crescent Pond | Hybrid |
| CP13B02 | This study | | Yes | Survivor | Martin & Gould 2020 | Crescent Pond | Hybrid |
| CP13B08 | This study | | Yes | Survivor | Martin & Gould 2020 | Crescent Pond | Hybrid |
| CP14C04 | This study | | Yes | Survivor | Martin & Gould 2020 | Crescent Pond | Hybrid |
| CP15A10 | This study | | Yes | Survivor | Martin & Gould 2020 | Crescent Pond | Hybrid |
| CP15B08 | This study | | Yes | Survivor | Martin & Gould 2020 | Crescent Pond | Hybrid |
| CP15E01 | This study | | Yes | Survivor | Martin & Gould 2020 | Crescent Pond | Hybrid |
| CP15E03 | This study | | Yes | Survivor | Martin & Gould 2020 | Crescent Pond | Hybrid |
| CP17A01 | This study | | Yes | Survivor | Martin & Gould 2020 | Crescent Pond | Hybrid |
| CP18B11 | This study | | Yes | Survivor | Martin & Gould 2020 | Crescent Pond | Hybrid |
| CP19B02 | This study | | Yes | Survivor | Martin & Gould 2020 | Crescent Pond | Hybrid |
| CP19C02 | This study | | Yes | Survivor | Martin & Gould 2020 | Crescent Pond | Hybrid |
| CP19C07 | This study | | Yes | Survivor | Martin & Gould 2020 | Crescent Pond | Hybrid |
| CP19F01 | This study | | Yes | Survivor | Martin & Gould 2020 | Crescent Pond | Hybrid |
| CP19G03 | This study | | Yes | Survivor | Martin & Gould 2020 | Crescent Pond | Hybrid |
| CPH01 | This study | | Yes | Survivor | Martin & Wainwright 2013 | Crescent Pond | Hybrid |
| CPH02 | This study | | Yes | Survivor | Martin & Wainwright 2013 | Crescent Pond | Hybrid |
| CPH03 | This study | | Yes | Survivor | Martin & Wainwright 2013 | Crescent Pond | Hybrid |
| CPH04 | This study | | Yes | Survivor | Martin & Wainwright 2013 | Crescent Pond | Hybrid |
| CPH05 | This study | | Yes | Survivor | Martin & Wainwright 2013 | Crescent Pond | Hybrid |
| CPH07 | This study | | Yes | Survivor | Martin & Wainwright 2013 | Crescent Pond | Hybrid |
| CPH08 | This study | | Yes | Survivor | Martin & Wainwright 2013 | Crescent Pond | Hybrid |
| CPH09 | This study | | Yes | Survivor | Martin & Wainwright 2013 | Crescent Pond | Hybrid |
| CPH10 | This study | | Yes | Survivor | Martin & Wainwright 2013 | Crescent Pond | Hybrid |
| CPH100 | This study | | Yes | Survivor | Martin & Wainwright 2013 | Crescent Pond | Hybrid |
| CPH11 | This study | | Yes | Survivor | Martin & Wainwright 2013 | Crescent Pond | Hybrid |
| CPH123 | This study | | Yes | Survivor | Martin & Wainwright 2013 | Crescent Pond | Hybrid |
| LL01H04 | This study | | Yes | Survivor | Martin & Gould 2020 | Little Lake | Hybrid |
| LL02G09 | This study | | Yes | Survivor | Martin & Gould 2020 | Little Lake | Hybrid |
| LL06B06 | This study | | Yes | Survivor | Martin & Gould 2020 | Little Lake | Hybrid |
| LL06E04 | This study | | Yes | Survivor | Martin & Gould 2020 | Little Lake | Hybrid |
| LL07E12 | This study | | Yes | Survivor | Martin & Gould 2020 | Little Lake | Hybrid |
| LL07G04 | This study | | Yes | Survivor | Martin & Gould 2020 | Little Lake | Hybrid |
| LL08A09 | This study | | Yes | Survivor | Martin & Gould 2020 | Little Lake | Hybrid |
| LL08B04 | This study | | Yes | Survivor | Martin & Gould 2020 | Little Lake | Hybrid |
| LL08D05 | This study | | Yes | Survivor | Martin & Gould 2020 | Little Lake | Hybrid |
| LL10F08 | This study | | Yes | Survivor | Martin & Gould 2020 | Little Lake | Hybrid |
| LL12B04 | This study | | Yes | Survivor | Martin & Gould 2020 | Little Lake | Hybrid |
| LL13B06 | This study | | Yes | Survivor | Martin & Gould 2020 | Little Lake | Hybrid |
| LL13D04 | This study | | Yes | Survivor | Martin & Gould 2020 | Little Lake | Hybrid |
| LL17H03 | This study | | Yes | Survivor | Martin & Gould 2020 | Little Lake | Hybrid |
| LL106 | This study | | Yes | Survivor | Martin & Wainwright 2013 | Little Lake | Hybrid |
| LL114 | This study | | Yes | Survivor | Martin & Wainwright 2013 | Little Lake | Hybrid |
| LL124 | This study | | Yes | Survivor | Martin & Wainwright 2013 | Little Lake | Hybrid |
| LL129 | This study | | Yes | Survivor | Martin & Wainwright 2013 | Little Lake | Hybrid |
| LL175 | This study | | Yes | Survivor | Martin & Wainwright 2013 | Little Lake | Hybrid |
| LL23 | This study | | Yes | Survivor | Martin & Wainwright 2013 | Little Lake | Hybrid |
| LL247 | This study | | Yes | Survivor | Martin & Wainwright 2013 | Little Lake | Hybrid |
| LL251 | This study | | Yes | Survivor | Martin & Wainwright 2013 | Little Lake | Hybrid |
| LL271 | This study | | Yes | Survivor | Martin & Wainwright 2013 | Little Lake | Hybrid |
| LLH12 | This study | | Yes | Survivor | Martin & Wainwright 2013 | Little Lake | Hybrid |
| LLH34 | This study | | Yes | Survivor | Martin & Wainwright 2013 | Little Lake | Hybrid |
| LLH41 | This study | | Yes | Survivor | Martin & Wainwright 2013 | Little Lake | Hybrid |
| LLH51 | This study | | Yes | Survivor | Martin & Wainwright 2013 | Little Lake | Hybrid |
| LLH94 | This study | | Yes | Survivor | Martin & Wainwright 2013 | Little Lake | Hybrid |
| CRPA1 | Richards et al. 2021 | | No | NA | NA | Crescent Pond | Generalist |
| CRPA1000 | Richards et al. 2021 | | No | NA | NA | Crescent Pond | Generalist |
| CRPA1001 | Richards et al. 2021 | | No | NA | NA | Crescent Pond | Generalist |
| CRPA1003 | Richards et al. 2021 | | No | NA | NA | Crescent Pond | Generalist |
| CRPA3 | Richards et al. 2021 | | No | NA | NA | Crescent Pond | Generalist |
| LILA1 | Richards et al. 2021 | | No | NA | NA | Little Lake | Generalist |
| OSPA1 | Richards et al. 2021 | | No | NA | NA | Osprey Pond (Little Lake) | Generalist |
| OSPA1000 | Richards et al. 2021 | | No | NA | NA | Osprey Pond (Little Lake) | Generalist |
| OSPA1001 | Richards et al. 2021 | | No | NA | NA | Osprey Pond (Little Lake) | Generalist |
| OSPA11 | Richards et al. 2021 | | No | NA | NA | Osprey Pond (Little Lake) | Generalist |
| OSPA12 | Richards et al. 2021 | | No | NA | NA | Osprey Pond (Little Lake) | Generalist |
| OSPA13 | Richards et al. 2021 | | No | NA | NA | Osprey Pond (Little Lake) | Generalist |
| OSPA4 | Richards et al. 2021 | | No | NA | NA | Osprey Pond (Little Lake) | Generalist |
| OSPA5 | Richards et al. 2021 | | No | NA | NA | Osprey Pond (Little Lake) | Generalist |
| OSPA6 | Richards et al. 2021 | | No | NA | NA | Osprey Pond (Little Lake) | Generalist |
| OSPA8 | Richards et al. 2021 | | No | NA | NA | Osprey Pond (Little Lake) | Generalist |
| OSPA9 | Richards et al. 2021 | | No | NA | NA | Osprey Pond (Little Lake) | Generalist |
| CRPM1 | Richards et al. 2021 | | No | NA | NA | Crescent Pond | Molluscivore |
| CRPM10 | Richards et al. 2021 | | No | NA | NA | Crescent Pond | Molluscivore |
| CRPM1000 | Richards et al. 2021 | | No | NA | NA | Crescent Pond | Molluscivore |
| CRPM1001 | Richards et al. 2021 | | No | NA | NA | Crescent Pond | Molluscivore |
| CRPM11 | Richards et al. 2021 | | No | NA | NA | Crescent Pond | Molluscivore |
| CRPM2 | Richards et al. 2021 | | No | NA | NA | Crescent Pond | Molluscivore |
| CRPM3 | Richards et al. 2021 | | No | NA | NA | Crescent Pond | Molluscivore |
| CRPM5 | Richards et al. 2021 | | No | NA | NA | Crescent Pond | Molluscivore |
| CRPM6 | Richards et al. 2021 | | No | NA | NA | Crescent Pond | Molluscivore |
| CRPM7 | Richards et al. 2021 | | No | NA | NA | Crescent Pond | Molluscivore |
| CRPM8 | Richards et al. 2021 | | No | NA | NA | Crescent Pond | Molluscivore |
| CRPM9 | Richards et al. 2021 | | No | NA | NA | Crescent Pond | Molluscivore |
| LILM-QTL | Richards et al. 2021 | | No | NA | NA | Little Lake | Molluscivore |
| LILM3 | Richards et al. 2021 | | No | NA | NA | Little Lake | Molluscivore |
| LILM4 | Richards et al. 2021 | | No | NA | NA | Little Lake | Molluscivore |
| LILM5 | Richards et al. 2021 | | No | NA | NA | Little Lake | Molluscivore |
| OSPM1 | Richards et al. 2021 | | No | NA | NA | Osprey Pond (Little Lake) | Molluscivore |
| OSPM1000 | Richards et al. 2021 | | No | NA | NA | Osprey Pond (Little Lake) | Molluscivore |
| OSPM1001 | Richards et al. 2021 | | No | NA | NA | Osprey Pond (Little Lake) | Molluscivore |
| OSPM11 | Richards et al. 2021 | | No | NA | NA | Osprey Pond (Little Lake) | Molluscivore |
| OSPM2 | Richards et al. 2021 | | No | NA | NA | Osprey Pond (Little Lake) | Molluscivore |
| OSPM3 | Richards et al. 2021 | | No | NA | NA | Osprey Pond (Little Lake) | Molluscivore |
| OSPM4 | Richards et al. 2021 | | No | NA | NA | Osprey Pond (Little Lake) | Molluscivore |
| OSPM5 | Richards et al. 2021 | | No | NA | NA | Osprey Pond (Little Lake) | Molluscivore |
| OSPM7 | Richards et al. 2021 | | No | NA | NA | Osprey Pond (Little Lake) | Molluscivore |
| OSPM8 | Richards et al. 2021 | | No | NA | NA | Osprey Pond (Little Lake) | Molluscivore |
| OSPM9 | Richards et al. 2021 | | No | NA | NA | Osprey Pond (Little Lake) | Molluscivore |
| CRPP-QTL | Richards et al. 2021 | | No | NA | NA | Crescent Pond | Scale-eater |
| CRPP1000 | Richards et al. 2021 | | No | NA | NA | Crescent Pond | Scale-eater |
| CRPP1001 | Richards et al. 2021 | | No | NA | NA | Crescent Pond | Scale-eater |
| CRPP2 | Richards et al. 2021 | | No | NA | NA | Crescent Pond | Scale-eater |
| CRPP3 | Richards et al. 2021 | | No | NA | NA | Crescent Pond | Scale-eater |
| CRPP4 | Richards et al. 2021 | | No | NA | NA | Crescent Pond | Scale-eater |
| CRPP5 | Richards et al. 2021 | | No | NA | NA | Crescent Pond | Scale-eater |
| CRPP7 | Richards et al. 2021 | | No | NA | NA | Crescent Pond | Scale-eater |
| CRPP8 | Richards et al. 2021 | | No | NA | NA | Crescent Pond | Scale-eater |
| CRPP9 | Richards et al. 2021 | | No | NA | NA | Crescent Pond | Scale-eater |
| LILP-QTL | Richards et al. 2021 | | No | NA | NA | Little Lake | Scale-eater |
| LILP3 | Richards et al. 2021 | | No | NA | NA | Little Lake | Scale-eater |
| LILP4 | Richards et al. 2021 | | No | NA | NA | Little Lake | Scale-eater |
| LILP5 | Richards et al. 2021 | | No | NA | NA | Little Lake | Scale-eater |
| OSPP1 | Richards et al. 2021 | | No | NA | NA | Osprey Pond (Little Lake) | Scale-eater |
| OSPP10 | Richards et al. 2021 | | No | NA | NA | Osprey Pond (Little Lake) | Scale-eater |
| OSPP1000 | Richards et al. 2021 | | No | NA | NA | Osprey Pond (Little Lake) | Scale-eater |
| OSPP1001 | Richards et al. 2021 | | No | NA | NA | Osprey Pond (Little Lake) | Scale-eater |
| OSPP11 | Richards et al. 2021 | | No | NA | NA | Osprey Pond (Little Lake) | Scale-eater |
| OSPP2 | Richards et al. 2021 | | No | NA | NA | Osprey Pond (Little Lake) | Scale-eater |
| OSPP3 | Richards et al. 2021 | | No | NA | NA | Osprey Pond (Little Lake) | Scale-eater |
| OSPP4 | Richards et al. 2021 | | No | NA | NA | Osprey Pond (Little Lake) | Scale-eater |
| OSPP5 | Richards et al. 2021 | | No | NA | NA | Osprey Pond (Little Lake) | Scale-eater |
| OSPP7 | Richards et al. 2021 | | No | NA | NA | Osprey Pond (Little Lake) | Scale-eater |
| OSPP9 | Richards et al. 2021 | | No | NA | NA | Osprey Pond (Little Lake) | Scale-eater |
| CPA01 | NA | | Yes | Parental | NA | Crescent Pond | Generalist |
| CPA03 | NA | | Yes | Parental | NA | Crescent Pond | Generalist |
| CPA05 | NA | | Yes | Parental | NA | Crescent Pond | Generalist |
| CPA07 | NA | | Yes | Parental | NA | Crescent Pond | Generalist |
| CPA09 | NA | | Yes | Parental | NA | Crescent Pond | Generalist |
| CPA11 | NA | | Yes | Parental | NA | Crescent Pond | Generalist |
| CPA13 | NA | | Yes | Parental | NA | Crescent Pond | Generalist |
| CPA15 | NA | | Yes | Parental | NA | Crescent Pond | Generalist |
| CPA17 | NA | | Yes | Parental | NA | Crescent Pond | Generalist |
| CPA19 | NA | | Yes | Parental | NA | Crescent Pond | Generalist |
| CPA21 | NA | | Yes | Parental | NA | Crescent Pond | Generalist |
| CPA23 | NA | | Yes | Parental | NA | Crescent Pond | Generalist |
| CPA25 | NA | | Yes | Parental | NA | Crescent Pond | Generalist |
| CPA27 | NA | | Yes | Parental | NA | Crescent Pond | Generalist |
| CPA29 | NA | | Yes | Parental | NA | Crescent Pond | Generalist |
| CPA31 | NA | | Yes | Parental | NA | Crescent Pond | Generalist |
| CPA33 | NA | | Yes | Parental | NA | Crescent Pond | Generalist |
| CPA35 | NA | | Yes | Parental | NA | Crescent Pond | Generalist |
| CPA37 | NA | | Yes | Parental | NA | Crescent Pond | Generalist |
| CPA39 | NA | | Yes | Parental | NA | Crescent Pond | Generalist |
| CPA41 | NA | | Yes | Parental | NA | Crescent Pond | Generalist |
| CPA43 | NA | | Yes | Parental | NA | Crescent Pond | Generalist |
| CPA45 | NA | | Yes | Parental | NA | Crescent Pond | Generalist |
| CPA47 | NA | | Yes | Parental | NA | Crescent Pond | Generalist |
| CPA49 | NA | | Yes | Parental | NA | Crescent Pond | Generalist |
| CPA51 | NA | | Yes | Parental | NA | Crescent Pond | Generalist |
| CPA53 | NA | | Yes | Parental | NA | Crescent Pond | Generalist |
| CPA55 | NA | | Yes | Parental | NA | Crescent Pond | Generalist |
| CPA57 | NA | | Yes | Parental | NA | Crescent Pond | Generalist |
| CPA59 | NA | | Yes | Parental | NA | Crescent Pond | Generalist |
| LLA20 | NA | | Yes | Parental | NA | Little Lake | Generalist |
| LLA21 | NA | | Yes | Parental | NA | Little Lake | Generalist |
| LLA22 | NA | | Yes | Parental | NA | Little Lake | Generalist |
| LLA23 | NA | | Yes | Parental | NA | Little Lake | Generalist |
| LLA24 | NA | | Yes | Parental | NA | Little Lake | Generalist |
| LLA25 | NA | | Yes | Parental | NA | Little Lake | Generalist |
| LLA26 | NA | | Yes | Parental | NA | Little Lake | Generalist |
| LLA27 | NA | | Yes | Parental | NA | Little Lake | Generalist |
| LLA28 | NA | | Yes | Parental | NA | Little Lake | Generalist |
| LLA29 | NA | | Yes | Parental | NA | Little Lake | Generalist |
| LLA30 | NA | | Yes | Parental | NA | Little Lake | Generalist |
| LLA31 | NA | | Yes | Parental | NA | Little Lake | Generalist |
| LLA32 | NA | | Yes | Parental | NA | Little Lake | Generalist |
| LLA33 | NA | | Yes | Parental | NA | Little Lake | Generalist |
| LLA34 | NA | | Yes | Parental | NA | Little Lake | Generalist |
| LLA35 | NA | | Yes | Parental | NA | Little Lake | Generalist |
| LLA36 | NA | | Yes | Parental | NA | Little Lake | Generalist |
| LLA37 | NA | | Yes | Parental | NA | Little Lake | Generalist |
| LLA38 | NA | | Yes | Parental | NA | Little Lake | Generalist |
| LLA39 | NA | | Yes | Parental | NA | Little Lake | Generalist |
| LLA40 | NA | | Yes | Parental | NA | Little Lake | Generalist |
| LLA41 | NA | | Yes | Parental | NA | Little Lake | Generalist |
| LLA42 | NA | | Yes | Parental | NA | Little Lake | Generalist |
| LLA43 | NA | | Yes | Parental | NA | Little Lake | Generalist |
| LLA44 | NA | | Yes | Parental | NA | Little Lake | Generalist |
| LLA45 | NA | | Yes | Parental | NA | Little Lake | Generalist |
| LLA46 | NA | | Yes | Parental | NA | Little Lake | Generalist |
| LLA47 | NA | | Yes | Parental | NA | Little Lake | Generalist |
| LLA48 | NA | | Yes | Parental | NA | Little Lake | Generalist |
| LLA49 | NA | | Yes | Parental | NA | Little Lake | Generalist |
| CPM01 | NA | | Yes | Parental | NA | Crescent Pond | Molluscivore |
| CPM02 | NA | | Yes | Parental | NA | Crescent Pond | Molluscivore |
| CPM03 | NA | | Yes | Parental | NA | Crescent Pond | Molluscivore |
| CPM04 | NA | | Yes | Parental | NA | Crescent Pond | Molluscivore |
| CPM05 | NA | | Yes | Parental | NA | Crescent Pond | Molluscivore |
| CPM06 | NA | | Yes | Parental | NA | Crescent Pond | Molluscivore |
| CPM07 | NA | | Yes | Parental | NA | Crescent Pond | Molluscivore |
| CPM08 | NA | | Yes | Parental | NA | Crescent Pond | Molluscivore |
| CPM09 | NA | | Yes | Parental | NA | Crescent Pond | Molluscivore |
| CPM10 | NA | | Yes | Parental | NA | Crescent Pond | Molluscivore |
| CPM11 | NA | | Yes | Parental | NA | Crescent Pond | Molluscivore |
| CPM12 | NA | | Yes | Parental | NA | Crescent Pond | Molluscivore |
| CPM13 | NA | | Yes | Parental | NA | Crescent Pond | Molluscivore |
| CPM14 | NA | | Yes | Parental | NA | Crescent Pond | Molluscivore |
| CPM15 | NA | | Yes | Parental | NA | Crescent Pond | Molluscivore |
| CPM16 | NA | | Yes | Parental | NA | Crescent Pond | Molluscivore |
| CPM17 | NA | | Yes | Parental | NA | Crescent Pond | Molluscivore |
| CPM18 | NA | | Yes | Parental | NA | Crescent Pond | Molluscivore |
| CPM19 | NA | | Yes | Parental | NA | Crescent Pond | Molluscivore |
| CPM20 | NA | | Yes | Parental | NA | Crescent Pond | Molluscivore |
| LLM01 | NA | | Yes | Parental | NA | Little Lake | Molluscivore |
| LLM02 | NA | | Yes | Parental | NA | Little Lake | Molluscivore |
| LLM03 | NA | | Yes | Parental | NA | Little Lake | Molluscivore |
| LLM04 | NA | | Yes | Parental | NA | Little Lake | Molluscivore |
| LLM05 | NA | | Yes | Parental | NA | Little Lake | Molluscivore |
| LLM06 | NA | | Yes | Parental | NA | Little Lake | Molluscivore |
| LLM07 | NA | | Yes | Parental | NA | Little Lake | Molluscivore |
| LLM08 | NA | | Yes | Parental | NA | Little Lake | Molluscivore |
| LLM09 | NA | | Yes | Parental | NA | Little Lake | Molluscivore |
| LLM10 | NA | | Yes | Parental | NA | Little Lake | Molluscivore |
| LLM11 | NA | | Yes | Parental | NA | Little Lake | Molluscivore |
| LLM12 | NA | | Yes | Parental | NA | Little Lake | Molluscivore |
| LLM13 | NA | | Yes | Parental | NA | Little Lake | Molluscivore |
| LLM14 | NA | | Yes | Parental | NA | Little Lake | Molluscivore |
| LLM15 | NA | | Yes | Parental | NA | Little Lake | Molluscivore |
| LLM16 | NA | | Yes | Parental | NA | Little Lake | Molluscivore |
| LLM17 | NA | | Yes | Parental | NA | Little Lake | Molluscivore |
| LLM18 | NA | | Yes | Parental | NA | Little Lake | Molluscivore |
| CPP01 | NA | | Yes | Parental | NA | Crescent Pond | Scale-eater |
| CPP02 | NA | | Yes | Parental | NA | Crescent Pond | Scale-eater |
| CPP03 | NA | | Yes | Parental | NA | Crescent Pond | Scale-eater |
| CPP04 | NA | | Yes | Parental | NA | Crescent Pond | Scale-eater |
| CPP05 | NA | | Yes | Parental | NA | Crescent Pond | Scale-eater |
| CPP06 | NA | | Yes | Parental | NA | Crescent Pond | Scale-eater |
| CPP07 | NA | | Yes | Parental | NA | Crescent Pond | Scale-eater |
| CPP08 | NA | | Yes | Parental | NA | Crescent Pond | Scale-eater |
| CPP09 | NA | | Yes | Parental | NA | Crescent Pond | Scale-eater |
| CPP10 | NA | | Yes | Parental | NA | Crescent Pond | Scale-eater |
| CPP11 | NA | | Yes | Parental | NA | Crescent Pond | Scale-eater |
| CPP12 | NA | | Yes | Parental | NA | Crescent Pond | Scale-eater |
| CPP13 | NA | | Yes | Parental | NA | Crescent Pond | Scale-eater |
| CPP14 | NA | | Yes | Parental | NA | Crescent Pond | Scale-eater |
| CPP15 | NA | | Yes | Parental | NA | Crescent Pond | Scale-eater |
| CPP16 | NA | | Yes | Parental | NA | Crescent Pond | Scale-eater |
| CPP17 | NA | | Yes | Parental | NA | Crescent Pond | Scale-eater |
| CPP18 | NA | | Yes | Parental | NA | Crescent Pond | Scale-eater |
| CPP19 | NA | | Yes | Parental | NA | Crescent Pond | Scale-eater |
| CPP20 | NA | | Yes | Parental | NA | Crescent Pond | Scale-eater |
| CPP21 | NA | | Yes | Parental | NA | Crescent Pond | Scale-eater |
| CPP22 | NA | | Yes | Parental | NA | Crescent Pond | Scale-eater |
| CPP23 | NA | | Yes | Parental | NA | Crescent Pond | Scale-eater |
| CPP24 | NA | | Yes | Parental | NA | Crescent Pond | Scale-eater |
| CPP25 | NA | | Yes | Parental | NA | Crescent Pond | Scale-eater |
| CPP26 | NA | | Yes | Parental | NA | Crescent Pond | Scale-eater |
| CPP27 | NA | | Yes | Parental | NA | Crescent Pond | Scale-eater |
| CPP28 | NA | | Yes | Parental | NA | Crescent Pond | Scale-eater |
| CPP29 | NA | | Yes | Parental | NA | Crescent Pond | Scale-eater |
| CPP30 | NA | | Yes | Parental | NA | Crescent Pond | Scale-eater |
| LLP01 | NA | | Yes | Parental | NA | Little Lake | Scale-eater |
| LLP02 | NA | | Yes | Parental | NA | Little Lake | Scale-eater |
| LLP03 | NA | | Yes | Parental | NA | Little Lake | Scale-eater |
| LLP04 | NA | | Yes | Parental | NA | Little Lake | Scale-eater |
| LLP05 | NA | | Yes | Parental | NA | Little Lake | Scale-eater |
| LLP06 | NA | | Yes | Parental | NA | Little Lake | Scale-eater |
| LLP07 | NA | | Yes | Parental | NA | Little Lake | Scale-eater |
| LLP08 | NA | | Yes | Parental | NA | Little Lake | Scale-eater |
| LLP09 | NA | | Yes | Parental | NA | Little Lake | Scale-eater |
| LLP10 | NA | | Yes | Parental | NA | Little Lake | Scale-eater |
| LLP11 | NA | | Yes | Parental | NA | Little Lake | Scale-eater |
| LLP12 | NA | | Yes | Parental | NA | Little Lake | Scale-eater |
| LLP13 | NA | | Yes | Parental | NA | Little Lake | Scale-eater |
| LLP14 | NA | | Yes | Parental | NA | Little Lake | Scale-eater |
| LLP15 | NA | | Yes | Parental | NA | Little Lake | Scale-eater |
| LLP16 | NA | | Yes | Parental | NA | Little Lake | Scale-eater |
| LLP17 | NA | | Yes | Parental | NA | Little Lake | Scale-eater |
| LLP18 | NA | | Yes | Parental | NA | Little Lake | Scale-eater |
| LLP19 | NA | | Yes | Parental | NA | Little Lake | Scale-eater |
| LLP20 | NA | | Yes | Parental | NA | Little Lake | Scale-eater |
| LLP21 | NA | | Yes | Parental | NA | Little Lake | Scale-eater |
| LLP22 | NA | | Yes | Parental | NA | Little Lake | Scale-eater |
| LLP23 | NA | | Yes | Parental | NA | Little Lake | Scale-eater |
| LLP24 | NA | | Yes | Parental | NA | Little Lake | Scale-eater |
| LLP25 | NA | | Yes | Parental | NA | Little Lake | Scale-eater |
| LLP26 | NA | | Yes | Parental | NA | Little Lake | Scale-eater |
| LLP27 | NA | | Yes | Parental | NA | Little Lake | Scale-eater |
| LLP28 | NA | | Yes | Parental | NA | Little Lake | Scale-eater |
| LLP29 | NA | | Yes | Parental | NA | Little Lake | Scale-eater |
| LLP30 | NA | | Yes | Parental | NA | Little Lake | Scale-eater |

**Supplementary file 1b**

**Supplementary file 1c**

**Supplementary file 1d**

**Supplementary file 1e**

**Supplementary file 1f**

| **Scaffold** | | **Position** | **Significance** | **REF** | **ALT** | **Variant Type** | **Gene Identifier** | **Gene Card** |
| --- | --- | --- | --- | --- | --- | --- | --- | --- |
| HiC_scaffold_1 | | 32071263 | FDR | A | C | intergenic | CBRO_00000660-CBRO_00000661 | Znf250-Ptgdr2 |
| **HiC_scaffold_1** | | **43866598** | **Bonferroni** | **G** | **A** | **intergenic** | **CBRO_00000910-CBRO_00000911** | **PPM1K-OVCH2** |
| HiC_scaffold_1 | | 43867614 | FDR | C | T | intergenic | CBRO_00000910-CBRO_00000911 | PPM1K-OVCH2 |
| HiC_scaffold_3 | | 2658774 | FDR | T | A | intergenic | CBRO_00017856-CBRO_00017857 | KMT2E-Magi2 |
| HiC_scaffold_3 | | 2658775 | FDR | T | C | intergenic | CBRO_00017856-CBRO_00017857 | KMT2E-Magi2 |
| HiC_scaffold_3 | | 2658793 | FDR | C | A | intergenic | CBRO_00017856-CBRO_00017857 | KMT2E-Magi2 |
| HiC_scaffold_4 | | 18899496 | FDR | T | C | intergenic | CBRO_00012427-CBRO_00012428 | UNKNOWN-edc4 |
| HiC_scaffold_5 | | 18306419 | FDR | C | G | upstream; intergenic | CBRO_00001232; CBRO_00001231-CBRO_00001232 | xlrs1; PPEF2-xlrs1 |
| HiC_scaffold_5 | | 18306428 | FDR | A | T | upstream; intergenic | CBRO_00001232; CBRO_00001231-CBRO_00001232 | xlrs1; PPEF2-xlrs1 |
| HiC_scaffold_5 | | 18307019 | FDR | G | T | intronic | CBRO_00001232 | xlrs1 |
| HiC_scaffold_5 | | 18307030 | FDR | G | A | intronic | CBRO_00001232 | xlrs1 |
| HiC_scaffold_5 | | 18311696 | FDR | C | T | intronic | CBRO_00001232 | xlrs1 |
| HiC_scaffold_5 | | 40475116 | FDR | T | A | intergenic | CBRO_00001627-CBRO_00001628 | SLC25A44-UBE2Q2 |
| HiC_scaffold_7 | | 10290141 | FDR | T | C | downstream; intergenic | CBRO_00009717; CBRO_00009716-CBRO_00009717 | Nfkbie; SLC35B2-Nfkbie |
| HiC_scaffold_7 | | 10290142 | FDR | G | C | downstream; intergenic | CBRO_00009717; CBRO_00009716-CBRO_00009717 | Nfkbie; SLC35B2-Nfkbie |
| HiC_scaffold_7 | | 10290165 | FDR | A | G | downstream; intergenic | CBRO_00009717; CBRO_00009716-CBRO_00009717 | Nfkbie; SLC35B2-Nfkbie |
| HiC_scaffold_7 | | 10290166 | FDR | T | C | downstream; intergenic | CBRO_00009717; CBRO_00009716-CBRO_00009717 | Nfkbie; SLC35B2-Nfkbie |
| HiC_scaffold_7 | | 10290168 | FDR | T | C | downstream; intergenic | CBRO_00009717; CBRO_00009716-CBRO_00009717 | Nfkbie; SLC35B2-Nfkbie |
| HiC_scaffold_7 | | 13815058 | FDR | A | G | synonymous; downstream | CBRO_00009805; CBRO_00009804 | Aloxe3; UNKNOWN |
| HiC_scaffold_7 | | 13815326 | FDR | C | T | intronic | CBRO_00009805 | Aloxe3 |
| HiC_scaffold_7 | | 15349830 | FDR | C | A | intergenic | CBRO_00009834-CBRO_00009835 | Adcy8-efr3b |
| HiC_scaffold_7 | | 18378061 | FDR | T | A | intergenic | CBRO_00009902-CBRO_00009903 | Fam84a-DDX1 |
| **HiC_scaffold_8** | | **20263964** | **Bonferroni** | **G** | **A** | **upstream; intergenic** | **CBRO_00010647; CBRO_00010647-CBRO_00010648** | **Srcin1; Srcin1-Srcin1** |
| HiC_scaffold_8 | | 31539262 | FDR | G | A | upstream; intergenic | CBRO_00010835; CBRO_00010834-CBRO_00010835 | Gjd3; Gjd3-Gjd3 |
| HiC_scaffold_11 | | 3106766 | FDR | T | A | intronic | CBRO_00013361 | prkdc |
| HiC_scaffold_11 | | 3138733 | FDR | G | A | intergenic | CBRO_00013361-CBRO_00013362 | prkdc-arhgap29 |
| HiC_scaffold_11 | | 5658921 | FDR | A | G | intronic | CBRO_00013404 | KAZN |
| HiC_scaffold_14 | | 17556007 | FDR | C | T | intergenic | CBRO_00014134-CBRO_00014135 | UNKNOWN-Abr |
| HiC_scaffold_14 | | 17556026 | FDR | C | A | intergenic | CBRO_00014134-CBRO_00014135 | UNKNOWN-Abr |
| HiC_scaffold_16 | | 32837191 | FDR | T | C | intronic | CBRO_00003226 | KIF1B |
| HiC_scaffold_16 | | 35727503 | FDR | G | C | intergenic | CBRO_00003289-CBRO_00003290 | Pip5k1c-Polr2e |
| HiC_scaffold_16 | | 40215889 | FDR | G | A | intergenic | CBRO_00003383-CBRO_00003384 | chst10-UNKNOWN |
| HiC_scaffold_16 | | 40300592 | FDR | C | A | intergenic | CBRO_00003384-CBRO_00003385 | UNKNOWN-Carmil3 |
| HiC_scaffold_18 | | 26969972 | FDR | T | G | intronic | CBRO_00013239 | CSAD |
| HiC_scaffold_18 | | 26970123 | FDR | C | T | synonymous | CBRO_00013239 | CSAD |
| HiC_scaffold_18 | | 26970601 | FDR | T | A | intronic | CBRO_00013239 | CSAD |
| HiC_scaffold_18 | | 26978410 | FDR | G | A | intronic | CBRO_00013240 | Znf740 |
| HiC_scaffold_20 | | 332642 | FDR | G | A | missense | CBRO_00016084 | GTF3C4 |
| HiC_scaffold_20 | | 332689 | FDR | A | G | missense | CBRO_00016084 | GTF3C4 |
| HiC_scaffold_20 | | 14820437 | FDR | A | T | intronic | CBRO_00016304 | MALT1 |
| HiC_scaffold_20 | | 14820448 | FDR | T | C | intronic | CBRO_00016304 | MALT1 |
| HiC_scaffold_24 | | 1469530 | FDR | G | T | intergenic | CBRO_00014375-CBRO_00014376 | UNKNOWN-UNKNOWN |
| HiC_scaffold_24 | | 3223583 | FDR | T | A | intergenic | CBRO_00014421-CBRO_00014422 | Gal3st3-RIN2 |
| HiC_scaffold_24 | | 11618442 | FDR | T | G | intronic | CBRO_00014601 | ABCA4 |
| **HiC_scaffold_24** | | **15964553** | **Bonferroni** | **C** | **T** | **intergenic** | **CBRO_00014635-CBRO_00014636** | **Lrfn2-SNX15** |
| HiC_scaffold_27 | | 1898180 | FDR | G | A | downstream; intergenic | CBRO_00005887; CBRO_00005886-CBRO_00005887 | UNKNOWN; hoxb13a-UNKNOWN |
| HiC_scaffold_27 | | 8137335 | FDR | G | T | intronic | CBRO_00005998 | SMARCA4 |
| HiC_scaffold_27 | | 9065056 | FDR | C | A | intergenic | CBRO_00006026-CBRO_00006027 | ANKFN1-ccdc134 |
| HiC_scaffold_27 | | 12370585 | FDR | C | T | intergenic | CBRO_00006131-CBRO_00006132 | SHISA9-Desi1 |
| HiC_scaffold_27 | | 32078665 | FDR | C | G | intergenic | CBRO_00006685-CBRO_00006686 | med25-Lrrc4b |
| HiC_scaffold_27 | | 34904388 | FDR | A | C | intergenic | CBRO_00006756-CBRO_00006757 | Grin2c-nog3 |
| HiC_scaffold_27 | | 35431570 | FDR | G | C | intergenic | CBRO_00006756-CBRO_00006757 | Grin2c-nog3 |
| HiC_scaffold_27 | | 35431578 | FDR | C | T | intergenic | CBRO_00006756-CBRO_00006757 | Grin2c-nog3 |
| HiC_scaffold_27 | | 35431585 | FDR | A | G | intergenic | CBRO_00006756-CBRO_00006757 | Grin2c-nog3 |
| HiC_scaffold_34 | | 16654675 | FDR | G | C | intergenic | CBRO_00001997-CBRO_00001998 | MDFIC2-foxp1b |
| HiC_scaffold_34 | | 19393244 | FDR | A | T | upstream; intergenic | CBRO_00002027; CBRO_00002027-CBRO_00002028 | SUOX; SUOX-SUOX |
| HiC_scaffold_34 | | 22010499 | FDR | C | T | intronic | CBRO_00002117 | GNAI2 |
| HiC_scaffold_34 | | 31220916 | FDR | T | A | upstream; intergenic | CBRO_00002389; CBRO_00002388-CBRO_00002389 | CTTNBP2NL; Kcnd3-CTTNBP2NL |
| HiC_scaffold_34 | | 37769304 | FDR | A | C | intergenic | CBRO_00002519-CBRO_00002520 | ASIC2-asic1 |
| HiC_scaffold_37 | | 5963405 | FDR | T | C | intergenic | CBRO_00011020-CBRO_00011021 | C14orf93-pim2 |
| HiC_scaffold_37 | | 11017168 | FDR | G | A | intergenic | CBRO_00011155-CBRO_00011156 | GALNT12-elp2 |
| HiC_scaffold_37 | | 13822135 | FDR | A | G | upstream; intergenic | CBRO_00011221; CBRO_00011220-CBRO_00011221 | SATB1; KCNH8-SATB1 |
| HiC_scaffold_37 | | 13823678 | FDR | G | A | upstream; intergenic | CBRO_00011221; CBRO_00011220-CBRO_00011221 | SATB1; KCNH8-SATB1 |
| HiC_scaffold_37 | | 13832007 | FDR | T | A | missense | CBRO_00011221 | SATB1 |
| HiC_scaffold_37 | | 16920863 | FDR | T | C | intergenic | CBRO_00011259-CBRO_00011260 | CSMD1-UNKNOWN |
| **HiC_scaffold_37** | | **18591438** | **Bonferroni** | **G** | **A** | **intergenic** | **CBRO_00011301-CBRO_00011302** | **cck-trim71** |
| HiC_scaffold_37 | | 18591463 | FDR | C | T | intergenic | CBRO_00011301-CBRO_00011302 | cck-trim71 |
| HiC_scaffold_37 | | 18596716 | FDR | A | T | intergenic | CBRO_00011301-CBRO_00011302 | cck-trim71 |
| HiC_scaffold_40 | | 5885291 | FDR | G | T | downstream; intergenic | CBRO_00016614; CBRO_00016613-CBRO_00016614 | C14orf93 homolog; HTR2A-C14orf93 homolog |
| HiC_scaffold_43 | | 2568535 | FDR | A | G | intergenic | CBRO_00008246-CBRO_00008247 | UNKNOWN-Gpr68 |
| HiC_scaffold_44 | | 25886134 | FDR | A | C | intergenic | CBRO_00007276-CBRO_00007277 | RAPGEF2-QDPR |
| HiC_scaffold_45 | | 885834 | FDR | A | C | upstream; intergenic | CBRO_00018766; CBRO_00018766-CBRO_00018767 | Mog; Mog-EPHB4 |
| HiC_scaffold_45 | | 2213441 | FDR | G | T | intronic | CBRO_00018814 | Nlrp12 |
| HiC_scaffold_46 | | 856248 | FDR | A | G | intergenic | CBRO_00007439-CBRO_00007440 | UNKNOWN-NLRP12 |
| HiC_scaffold_46 | | 1232350 | FDR | G | A | intronic | CBRO_00007448 | NEB |
| HiC_scaffold_46 | | 30183758 | FDR | C | G | intergenic | CBRO_00008059-CBRO_00008060 | TFRC-Rgs11 |
| HiC_scaffold_46 | | 32048848 | FDR | A | C | intronic | CBRO_00008105 | Hsd17b7 |
| HiC_scaffold_46 | | 32050109 | FDR | G | T | synonymous | CBRO_00008105 | Hsd17b7 |
| **HiC_scaffold_46** | | **35151009** | **Bonferroni** | **T** | **C** | **downstream; intergenic** | **CBRO_00008165; CBRO_00008165-CBRO_00008166** | **Klf9; Klf9-Tsen15** |
| HiC_scaffold_46 | | 35163681 | FDR | T | C | downstream; downstream; intergenic | CBRO_00008166; CBRO_00008167; CBRO_00008166-CBRO_00008167 | Tsen15; UNKNOWN; Tsen15-UNKNOWN |
| HiC_scaffold_46 | | 35164267 | FDR | A | G | downstream; downstream; intergenic | CBRO_00008166; CBRO_00008167; CBRO_00008166-CBRO_00008167 | Tsen15; UNKNOWN; Tsen15-UNKNOWN |
| HiC_scaffold_47 | | 787141 | FDR | T | G | upstream; intergenic | CBRO_00008838; CBRO_00008837-CBRO_00008838 | Nlrc3; NLRC3-Nlrc3 |
| HiC_scaffold_47 | | 4300295 | FDR | G | A | downstream; intergenic | CBRO_00008933; CBRO_00008933-CBRO_00008934 | NEK6; NEK6-Psmb7 |
| HiC_scaffold_47 | | 6767766 | FDR | T | C | intergenic | CBRO_00008991-CBRO_00008992 | TACR1-Grk5 |
| HiC_scaffold_52 | | 20791197 | FDR | T | A | upstream; intronic | CBRO_00011892; CBRO_00011891 | rabl3; GTF2E1 |
| HiC_scaffold_52 | | 22551701 | FDR | G | C | intergenic | CBRO_00011922-CBRO_00011923 | ALS2-Serp2 |
| HiC_scaffold_52 | | 31021517 | FDR | C | T | intergenic | CBRO_00012051-CBRO_00012052 | Tmeff2-slc39a10 |
| **HiC_scaffold_53** | | **11317840** | **Bonferroni** | **A** | **G** | **intergenic** | **CBRO_00005178-CBRO_00005179** | **Fucolectin-1-Fucolectin-5** |
| HiC_scaffold_53 | | 11326035 | FDR | C | T | intergenic | CBRO_00005178-CBRO_00005179 | Fucolectin-1-Fucolectin-5 |
| HiC_scaffold_53 | | 11326410 | FDR | G | A | intergenic | CBRO_00005178-CBRO_00005179 | Fucolectin-1-Fucolectin-5 |
| HiC_scaffold_53 | | 11331079 | FDR | A | G | intergenic | CBRO_00005178-CBRO_00005179 | Fucolectin-1-Fucolectin-5 |
| HiC_scaffold_53 | | 15966447 | FDR | G | A | upstream; downstream; intergenic | CBRO_00005235; CBRO_00005234; CBRO_00005234-CBRO_00005235 | Tmem222; WDTC1; WDTC1-Tmem222 |
| HiC_scaffold_53 | | 17413090 | FDR | A | G | missense | CBRO_00005274 | Mag |
| HiC_scaffold_53 | | 20715576 | FDR | C | A | intergenic | CBRO_00005380-CBRO_00005381 | Scrt2-Ino80c |
| HiC_scaffold_53 | | 20715623 | FDR | G | A | intergenic | CBRO_00005380-CBRO_00005381 | Scrt2-Ino80c |
| HiC_scaffold_53 | | 20715851 | FDR | A | G | intergenic | CBRO_00005380-CBRO_00005381 | Scrt2-Ino80c |
| HiC_scaffold_53 | | 27386094 | FDR | G | T | intronic | CBRO_00005625 | Arhgef1 |
| HiC_scaffold_53 | | 27396961 | FDR | T | A | intronic | CBRO_00005625 | Arhgef1 |
| HiC_scaffold_53 | | 27398605 | FDR | T | G | downstream; intergenic | CBRO_00005625; CBRO_00005625-CBRO_00005626 | Arhgef1; Arhgef1-CD79A |
| HiC_scaffold_53 | | 33282501 | FDR | A | G | intergenic | CBRO_00005732-CBRO_00005733 | mios-GLCCI1 |
| HiC_scaffold_53 | | 39228193 | FDR | T | C | intergenic | CBRO_00005830-CBRO_00005831 | UNKNOWN-GnRHR2 |
| **HiC_scaffold_53** | | **39228264** | **Bonferroni** | **C** | **T** | **intergenic** | **CBRO_00005830-CBRO_00005831** | **UNKNOWN-GnRHR2** |
| HiC_scaffold_53 | | 39228942 | FDR | A | T | intergenic | CBRO_00005830-CBRO_00005831 | UNKNOWN-GnRHR2 |
| HiC_scaffold_53 | | 39279263 | FDR | G | T | intronic | CBRO_00005832 | IGDCC3 |
| HiC_scaffold_53 | | 39770914 | FDR | A | T | intronic | CBRO_00005849 | Cpne4 |
| HiC_scaffold_611 | | 5621 | FDR | A | T | intergenic | CHR_START-CBRO_00020243 | CHR_START-fzdz-a |
| HiC_scaffold_611 | | 5625 | FDR | G | T | intergenic | CHR_START-CBRO_00020243 | CHR_START-fzdz-a |
| HiC_scaffold_611 | | 5634 | FDR | T | A | intergenic | CHR_START-CBRO_00020243 | CHR_START-fzdz-a |
| HiC_scaffold_1053 | | 3494 | FDR | A | G | downstream; intergenic | CBRO_00020503; CHR_START-CBRO_00020503 | UBE2G2; CHR_START-UBE2G2 |
| HiC_scaffold_1133 | | 9946 | FDR | A | G | intergenic | . | . |
| HiC_scaffold_1371 | | 7318 | FDR | C | T | upstream; intergenic | CBRO_00021026; CHR_START-CBRO_00021026 | UNKNOWN; CHR_START-UNKNOWN |
| **HiC_scaffold_1848** | | **40119** | **Bonferroni** | **C** | **A** | **intergenic** | **.** | **.** |
| **HiC_scaffold_1848** | | **40465** | **Bonferroni** | **T** | **A** | **intergenic** | **.** | **.** |
| **HiC_scaffold_1848** | | **40590** | **Bonferroni** | **T** | **C** | **intergenic** | **.** | **.** |
| HiC_scaffold_1848 | | 40877 | FDR | T | C | intergenic | . | . |
| HiC_scaffold_1848 | | 41351 | FDR | T | C | intergenic | . | . |
| HiC_scaffold_2220 | | 10128 | FDR | G | T | intergenic | . | . |
| **HiC_scaffold_4461** | | **12939** | **Bonferroni** | **T** | **A** | **intergenic** | **.** | **.** |
| HiC_scaffold_4665 | | 13941 | FDR | C | T | intergenic | . | . |
| HiC_scaffold_6275 | | 6000 | FDR | C | A | intergenic | . | . |
| HiC_scaffold_6337 | | 5745 | FDR | T | G | intronic | CBRO_00021217 | PKP3 |
| HiC_scaffold_6769 | | 2796 | FDR | G | A | intergenic | . | . |
| HiC_scaffold_6963 | | 5970 | FDR | C | A | intergenic | . | . |
| HiC_scaffold_6963 | | 6101 | FDR | A | G | intergenic | . | . |
| HiC_scaffold_9280 | | 3448 | FDR | G | A | intergenic | . | . |
| HiC_scaffold_9949 | | 52 | FDR | C | T | intergenic | . | . |
| HiC_scaffold_10928 | | 3575 | FDR | G | A | downstream; intergenic | CBRO_00021896; CHR_START-CBRO_00021896 | CYP2A10; CHR_START-CYP2A10 |
| HiC_scaffold_11921 | | 5560 | FDR | A | T | intergenic | . | . |
| HiC_scaffold_12068 | | 2929 | FDR | G | T | intergenic | . | . |
| **HiC_scaffold_12778** | **1456** | | **Bonferroni** | **T** | **A** | **missense** | **CBRO_00022026** | **COL8A1** |
| HiC_scaffold_17578 | | 180 | FDR | T | G | intergenic | . | . |
| **HiC_scaffold_18999** | | **1084** | **Bonferroni** | **A** | **G** | **intergenic** | **.** | **.** |

**Supplementary file 1g**

**Supplementary file 1h**

| **Scaffold** | **Position** | **Significance** | **REF** | **ALT** | **Variant Type** | **Gene Identifier** | **Gene Card** |
| --- | --- | --- | --- | --- | --- | --- | --- |
| HiC_scaffold_4 | 8897671 | FDR | A | T | intergenic | CBRO_00012254-CBRO_00012255 | Megf10-UNKNOWN |
| HiC_scaffold_4 | 16057698 | FDR | C | A | intergenic | CBRO_00012386-CBRO_00012387 | CCND2-Mlycd |
| HiC_scaffold_4 | 29273446 | FDR | T | C | synonymous | CBRO_00012620 | CKAP5 |
| HiC_scaffold_4 | 29273458 | FDR | C | T | synonymous | CBRO_00012620 | CKAP5 |
| HiC_scaffold_5 | 18259775 | FDR | C | A | upstream; intergenic | CBRO_00001229; CBRO_00001229-CBRO_00001230 | AP1S2; AP1S2-phka2 |
| *HiC_scaffold_5* | *18306419* | *FDR* | *C* | *G* | *upstream; intergenic* | *CBRO_00001232; CBRO_00001231-CBRO_00001232* | *xlrs1; PPEF2-xlrs1* |
| *HiC_scaffold_5* | *18306428* | *FDR* | *A* | *T* | *upstream; intergenic* | *CBRO_00001232; CBRO_00001231-CBRO_00001232* | *xlrs1; PPEF2-xlrs1* |
| *HiC_scaffold_5* | *18307019* | *FDR* | *G* | *T* | *intronic* | *CBRO_00001232* | *xlrs1* |
| *HiC_scaffold_5* | *18307030* | *FDR* | *G* | *A* | *intronic* | *CBRO_00001232* | *xlrs1* |
| HiC_scaffold_5 | 36619253 | FDR | T | C | intergenic | CBRO_00001583-CBRO_00001584 | Chst12-ZDHHC13 |
| *HiC_scaffold_7* | *13815058* | *FDR* | *A* | *G* | *synonymous; downstream* | *CBRO_00009805; CBRO_00009804* | *Aloxe3; UNKNOWN* |
| HiC_scaffold_7 | 13823565 | FDR | T | C | intronic | CBRO_00009806 | Fbxo30 |
| HiC_scaffold_7 | 13824467 | FDR | A | G | missense | CBRO_00009806 | Fbxo30 |
| HiC_scaffold_7 | 19371997 | FDR | C | T | upstream; intergenic | CBRO_00009922; CBRO_00009921-CBRO_00009922 | ELOVL4; TENT5A-ELOVL4 |
| HiC_scaffold_7 | 19372002 | FDR | T | G | upstream; intergenic | CBRO_00009922; CBRO_00009921-CBRO_00009922 | ELOVL4; TENT5A-ELOVL4 |
| HiC_scaffold_8 | 20265076 | FDR | T | C | upstream; intergenic | CBRO_00010647; CBRO_00010647-CBRO_00010648 | Srcin1; Srcin1-Srcin1 |
| HiC_scaffold_8 | 20265098 | FDR | G | C | upstream; intergenic | CBRO_00010647; CBRO_00010647-CBRO_00010648 | Srcin1; Srcin1-Srcin1 |
| HiC_scaffold_8 | 20278571 | FDR | G | A | intergenic | CBRO_00010647-CBRO_00010648 | Srcin1-Srcin1 |
| HiC_scaffold_9 | 15585466 | FDR | C | G | missense | CBRO_00004552 | Tmem260 |
| HiC_scaffold_9 | 18453213 | FDR | A | G | intergenic | CBRO_00004639-CBRO_00004640 | Bub1b-PAK6 |
| HiC_scaffold_9 | 28127377 | FDR | C | T | intergenic | CBRO_00004857-CBRO_00004858 | METTL21E-RASA3 |
| HiC_scaffold_10 | 193812 | FDR | C | G | intergenic | CBRO_00018931-CBRO_00018932 | Spsb4-UNKNOWN |
| HiC_scaffold_11 | 25632195 | FDR | C | T | intergenic | CBRO_00013717-CBRO_00013718 | CDH10-Cdh6 |
| HiC_scaffold_11 | 25632258 | FDR | C | T | intergenic | CBRO_00013717-CBRO_00013718 | CDH10-Cdh6 |
| HiC_scaffold_11 | 25632641 | FDR | A | G | intergenic | CBRO_00013717-CBRO_00013718 | CDH10-Cdh6 |
| HiC_scaffold_14 | 14624430 | FDR | T | C | intergenic | CBRO_00014084-CBRO_00014085 | KDM6B-FGF11 |
| HiC_scaffold_18 | 26970449 | FDR | C | T | intronic | CBRO_00013239 | CSAD |
| *HiC_scaffold_27* | *8137335* | *FDR* | *G* | *T* | *intronic* | *CBRO_00005998* | *SMARCA4* |
| HiC_scaffold_27 | 21919164 | FDR | G | T | synonymous | CBRO_00006396 | SSTR2 |
| HiC_scaffold_29 | 2136546 | FDR | A | T | upstream; intergenic | CBRO_00016347; CBRO_00016347-CBRO_00016348 | RAPGEF6; RAPGEF6-ACSL6 |
| HiC_scaffold_29 | 2147361 | FDR | C | T | intergenic | CBRO_00016347-CBRO_00016348 | RAPGEF6-ACSL6 |
| HiC_scaffold_31 | 6226140 | FDR | A | C | intronic | CBRO_00017224 | ranbp9 |
| HiC_scaffold_34 | 7439419 | FDR | G | T | intergenic | CBRO_00001828-CBRO_00001829 | rnf152-CAMTA1 |
| HiC_scaffold_34 | 30571660 | FDR | C | A | intronic | CBRO_00002374 | SHMT2 |
| HiC_scaffold_40 | 4693917 | FDR | G | A | intronic | CBRO_00016589 | SLC37A1 |
| HiC_scaffold_40 | 4694015 | FDR | T | C | synonymous | CBRO_00016589 | SLC37A1 |
| HiC_scaffold_40 | 4723358 | FDR | A | G | intronic | CBRO_00016591 | UBXN4 |
| HiC_scaffold_40 | 4732538 | FDR | T | C | intronic | CBRO_00016591 | UBXN4 |
| HiC_scaffold_40 | 4734825 | FDR | G | T | intronic | CBRO_00016591 | UBXN4 |
| HiC_scaffold_40 | 4780518 | FDR | G | C | intronic | CBRO_00016592 | ITGB2 |
| HiC_scaffold_40 | 5016538 | FDR | C | A | upstream; intergenic | CBRO_00016599; CBRO_00016599-CBRO_00016600 | RRP1B; RRP1B-ITGB2 |
| HiC_scaffold_43 | 26869231 | FDR | C | G | synonymous | CBRO_00008677 | lrpprc |
| HiC_scaffold_43 | 27800569 | FDR | C | T | downstream; intergenic | CBRO_00008685; CBRO_00008685-CBRO_00008686 | timp3; timp3-ETV6 |
| HiC_scaffold_44 | 19344526 | FDR | C | T | intergenic | CBRO_00007178-CBRO_00007179 | ATP11C-sox3 |
| HiC_scaffold_46 | 16495639 | FDR | G | A | intronic | CBRO_00007743 | PHLPP1 |
| HiC_scaffold_46 | 16510323 | FDR | G | A | intronic | CBRO_00007743 | PHLPP1 |
| HiC_scaffold_46 | 16512668 | FDR | T | A | intronic | CBRO_00007743 | PHLPP1 |
| **HiC_scaffold_46** | **16512886** | **Bonferroni** | **T** | **A** | **intronic** | **CBRO_00007743** | **PHLPP1** |
| HiC_scaffold_46 | 16513809 | FDR | T | C | synonymous | CBRO_00007743 | PHLPP1 |
| HiC_scaffold_52 | 19031083 | FDR | T | A | intergenic | CBRO_00011872-CBRO_00011873 | NRP2-MREG |
| HiC_scaffold_52 | 19031216 | FDR | A | T | intergenic | CBRO_00011872-CBRO_00011873 | NRP2-MREG |
| ***HiC_scaffold_1848*** | ***40119*** | ***Bonferroni*** | ***C*** | ***A*** | ***intergenic*** | ***.*** | ***.*** |
| ***HiC_scaffold_1848*** | ***40465*** | ***Bonferroni*** | ***T*** | ***A*** | ***intergenic*** | ***.*** | ***.*** |
| *HiC_scaffold_1848* | *40590* | *FDR* | *T* | *C* | *intergenic* | *.* | *.* |
| *HiC_scaffold_1848* | *40877* | *FDR* | *T* | *C* | *intergenic* | *.* | *.* |
| *HiC_scaffold_1848* | *41351* | *FDR* | *T* | *C* | *intergenic* | *.* | *.* |
| **HiC_scaffold_7644** | **5971** | **Bonferroni** | **T** | **G** | **intergenic** | **.** | **.** |
| HiC_scaffold_12681 | 4019 | FDR | T | C | upstream; intergenic | CBRO_00022068; CBRO_00022068-CHR_END | UNKNOWN; UNKNOWN-CHR_END |

**Supplementary file 1i**

**Supplementary file 1j**

| **Trait Index** | **Trait Description** | **Trait Shorthand** | **Points** |
| --- | --- | --- | --- |
| 1 | Nasal protrusion | nose | 3-4 |
| 2 | Nasal length | foresnout | 2-5 |
| 3 | Orbit to anal fin insertion | bellylen | 6-15 |
| 4 | Lateral facial length | snoutlen | 2-6 |
| 5 | Upper jaw to pectoral girdle | jaw2pect | 2-14 |
| 6 | Lateral skull length | pmx2add | 2-11 |
| 7 | Premaxilla length | pmxlen | 2-9 |
| 8 | Lower mandible length | jawlen | 1-9 |
| 9 | Jaw joint to orbit | foreeyewidth | 6-9 |
| 10 | Horizontal orbit diameter | eyewidth | 6-8 |
| 11 | Vertical orbit diameter | eyeht | 7-10 |
| 12 | Head height | headht | 7-9 |
| 13 | Suspensorium length | suspensorium | 9-11 |
| 14 | Adductor height | adductorht | 11-12 |
| 15 | Subopercle to pectoral girdle | ad2pect | 11-14 |
| 16 | Pectoral fin insertion width | pectinsertion | 13-14 |
| 17 | Anal to caudal distance | analtocaudal | 15-16 |
| 18 | Caudal peduncle height | caudalpedht | 16-18 |
| 19 | Dorsal to caudal distance | dorsaltocaudal | 18-19 |
| 20 | Body depth | bodydepth | 15-19 |
| 21 | Nasal protrusion angle | nasalangle | 7-5-3 |
| 22 | Premaxilla to orbit angle | topeyeangle | 7-2-10 |
| 23 | Premaxilla to adductor angle | lowereyeangle | 7-2-11 |
| 24 | Dorsal facial length | dorsalsnoutlen | 23-24, 25-26 |
| 25 | Adductor to premaxilla | eyetosnout | 21-24, 25-28 |
| 26 | Neurocranium to premaxilla | headlen | 24-20, 25-29 |
| 27 | Orbit to premaxilla | innereyetosnout | 22-24, 25-27 |
| 28 | Interorbital width | cranialwidth | 22-27 |
| 29 | Orbital neurocranium width | hindeyewidth | 21-28 |
| 30 | Max. neurocranium width | headwidth | 20-29 |
| 31 | Standard length (SL) | SL | 2-17 |
|  |  |  |  |

**Supplementary file 1k**

| **Model** | **AICc** | **ΔAICc** | **Akaike Weights** |
| --- | --- | --- | --- |
| *Composite ~ s(LD1, LD2) + Experiment + Lake* | *99.114* | *0.000* | *0.825* |
| Composite ~ s(LD1, LD2) + Experiment * Lake | 102.210 | 3.096 | 0.175 |
| Composite ~ s(LD1, LD2) + s(LD1) + s(LD2) + Experiment + Lake | 131.456 | 32.342 | < 0.001 |
| Composite ~ s(LD1, LD2) + s(LD1) + s(LD2) + Experiment * Lake | 135.894 | 36.781 | < 0.001 |
| Composite ~ s(LD1, LD2) + s(LD1, Experiment, bs = "fs") + s(LD2, Experiment, bs = "fs") + Lake | 230.428 | 131.314 | < 0.001 |
| Composite ~ s(LD1, LD2) + s(LD1, Lake, bs = "fs") + s(LD2, Lake, bs = "fs") + Experiment | 230.868 | 131.754 | < 0.001 |

**Supplementary file 1l**

| **Model** | **AICc** | **ΔAICc** | **Akaike Weights** |
| --- | --- | --- | --- |
| *Growth ~ s(LD1, LD2) + Experiment * Lake* | *-44.658* | *0.000* | *1* |
| Growth ~ s(LD1, LD2) + Experiment + Lake | 3.904 | 48.562 | < 0.001 |
| Growth ~ s(LD1, LD2) + s(LD1) + s(LD2) + Experiment * Lake | 46.249 | 90.907 | < 0.001 |
| Growth ~ s(LD1, LD2) + s(LD1) + s(LD2) + Experiment + Lake | 89.121 | 133.779 | < 0.001 |
| Growth ~ s(LD1, LD2) + s(LD1, Lake, bs = "fs") + s(LD2, Lake, bs = "fs") + Experiment | 690.379 | 735.038 | < 0.001 |
| Growth ~ s(LD1, LD2) + s(LD1, Experiment, bs = "fs") + s(LD2, Experiment, bs = "fs") + Lake | 693.748 | 738.406 | < 0.001 |

**Supplementary file 1m**

| **Model** | **AICc** | **ΔAICc** | **Akaike Weights** |
| --- | --- | --- | --- |
| *Survival ~ s(LD1, LD2) + Experiment + Lake* | *141.057* | *0.000* | *0.849* |
| Survival ~ s(LD1, LD2) + Experiment * Lake | 144.504 | 3.447 | 0.151 |
| Survival ~ s(LD1, LD2) + s(LD1) + s(LD2) + Experiment + Lake | 173.399 | 32.342 | < 0.001 |
| Survival ~ s(LD1, LD2) + s(LD1) + s(LD2) + Experiment * Lake | 178.189 | 37.132 | < 0.001 |
| Survival ~ s(LD1, LD2) + s(LD1, Experiment, bs = "fs") + s(LD2, Experiment, bs = "fs") + Lake | 273.547 | 132.490 | < 0.001 |
| Survival ~ s(LD1, LD2) + s(LD1, Lake, bs = "fs") + s(LD2, Lake, bs = "fs") + Experiment | 273.694 | 132.637 | < 0.001 |

**Supplementary file 1n**

| **Model** | **AICc** | **ΔAICc** | **Akaike Weights** |
| --- | --- | --- | --- |
| *Composite Fitness ~ s(LD1, LD2) + Experiment + Lake + s(Site1) + s(Site2) + s(Site6) + s(Site7) + s(Site8) + s(Site9) + s(Site10)* | *4.586* | *0.000* | *0.999* |
| Composite Fitness ~ s(LD1, LD2) + Experiment + Lake + s(Site1) + s(Site2) + s(Site3) + s(Site4) + s(Site5) + s(Site6) + s(Site7) + s(Site8) + s(Site9) + s(Site10) | 40.876 | 36.290 | < 0.001 |
| Composite Fitness ~ s(LD1, LD2) + Experiment + Lake + s(Site3) | 55.588 | 51.001 | < 0.001 |
| Composite Fitness ~ s(LD1, LD2) + Experiment + Lake + s(Site7) | 58.386 | 53.800 | < 0.001 |
| Composite Fitness ~ s(LD1, LD2) + Experiment + Lake + s(Site4) | 65.453 | 60.867 | < 0.001 |
| Composite Fitness ~ s(LD1, LD2) + Experiment + Lake + s(Site2) | 71.245 | 66.658 | < 0.001 |
| Composite Fitness ~ s(LD1, LD2) + Experiment + Lake + s(Site5) | 72.329 | 67.743 | < 0.001 |
| Composite Fitness ~ s(LD1, LD2) + Experiment + Lake + s(Site1) | 73.671 | 69.085 | < 0.001 |
| Composite Fitness ~ s(LD1, LD2) + Experiment + Lake + s(Site8) | 74.413 | 69.827 | < 0.001 |
| Composite Fitness ~ s(LD1, LD2) + Experiment + Lake + s(Site9) | 74.680 | 70.094 | < 0.001 |
| Composite Fitness ~ s(LD1, LD2) + Experiment + Lake + s(Site10) | 88.977 | 84.391 | < 0.001 |
| Composite Fitness ~ s(LD1, LD2) + Experiment + Lake + s(Site6) | 90.427 | 85.841 | < 0.001 |
| Composite Fitness ~ s(LD1, LD2) + Experiment + Lake | 99.114 | 94.527 | < 0.001 |

Note: Site1 = HiC_Scaffold_1:43866598, Site2 = HiC_Scaffold_53:11317840, Site3 = HiC_Scaffold_46:35151009, Site4 = HiC_Scaffold_8:20263964, Site5 = 37:18591438, Site5 = HiC_Scaffold_37:18591438, Site6 = HiC_Scaffold_24:15964553, Site7 = HiC_Scaffold_1848:40590, Site8 = HiC_Scaffold_4461:12939, Site9 = HiC_Scaffold_12778:1456, Site10 = HiC_Scaffold_18999:1084.

**Supplementary file 1o**

| **Model** | **AICc** | **ΔAICc** | **Akaike Weights** |
| --- | --- | --- | --- |
| Growth ~ s(LD1, LD2) + Experiment * Lake + s(Site3) + s(Site4) | -67.649 | 0.000 | 0.490 |
| Growth ~ s(LD1, LD2) + Experiment * Lake + s(Site3) | -65.634 | 2.015 | 0.179 |
| Growth ~ s(LD1, LD2) + Experiment * Lake + s(Site1) | -64.161 | 3.488 | 0.086 |
| Growth ~ s(LD1, LD2) + Experiment * Lake + s(Site1) + s(Site3) | -63.926 | 3.723 | 0.076 |
| Growth ~ s(LD1, LD2) + Experiment * Lake + s(Site1) + s(Site2) | -63.861 | 3.788 | 0.074 |
| Growth ~ s(LD1, LD2) + Experiment * Lake + s(Site2) + s(Site4) | -63.503 | 4.146 | 0.062 |
| Growth ~ s(LD1, LD2) + Experiment * Lake + s(Site2) | -61.849 | 5.800 | 0.027 |
| Growth ~ s(LD1, LD2) + Experiment * Lake + s(Site1) + s(Site4) | -58.044 | 9.604 | 0.004 |
| Growth ~ s(LD1, LD2) + Experiment * Lake + s(Site4) | -56.068 | 11.581 | 0.001 |
| Growth ~ s(LD1, LD2) + Experiment * Lake + s(Site1) + s(Site3) + s(Site4) | -54.878 | 12.770 | < 0.001 |
| Growth ~ s(LD1, LD2) + Experiment * Lake + s(Site1) + s(Site2) + s(Site4) | -54.509 | 13.140 | < 0.001 |
| Growth ~ s(LD1, LD2) + Experiment * Lake + s(Site2) + s(Site3) | -47.602 | 20.047 | < 0.001 |
| Growth ~ s(LD1, LD2) + Experiment * Lake | -44.658 | 22.990 | < 0.001 |
| Growth ~ s(LD1, LD2) + Experiment * Lake + s(Site1) + s(Site2) + s(Site3) | -41.689 | 25.960 | < 0.001 |
| Growth ~ s(LD1, LD2) + Experiment * Lake + s(Site1) + s(Site2) + s(Site3) + s(Site4) | -29.801 | 37.847 | < 0.001 |

Note: Site1 = HiC_Scaffold_46:16512886, Site2 = HiC_Scaffold_1848:40119, Site3 = HiC_Scaffold_1848:40465, Site4 = HiC_Scaffold_7644:5971

**Supplementary file 1p**

**Supplementary file 1q**

| **Comparison** | **Summary Statistic** | **Mean / SE** | **Mean / SE** | **Odds Ratio: (95% CI)** | **LRT *P*-value** |
| --- | --- | --- | --- | --- | --- |
|  |  | *Molluscivore Network* | *Scale-Eater Network* | *Molluscivore / Scale Eater* |  |
| **Generalist to Specialist** | *Number of nodes in network* | 22.994 / 0.106 | 31.000 / 0.177 | 0.818: (0.807, 0.829) | < 0.0001 |
|  | *Number of accessible paths* | 1.105 / 0.007 | 1.268 / 0.018 | 0.515: (0.449, 0.588) | < 0.0001 |
|  | *Scaled number of accessible paths* | 0.051 / 0.001 | 0.042 / 0.001 | 2.095: (1.934, 2.274) | < 0.0001 |
|  | *Length of shortest accessible path* | 2.410 / 0.012 | 3.444 / 0.029 | 0.253: (0.231, 0.277) | < 0.0001 |
| **Peaks in Network** | *Number of peaks* | 3.274 / 0.035 | 4.637 / 0.046 | 0.604: (0.575, 0.634) | < 0.0001 |
|  | *Scaled number of accessible paths to peaks* | 0.095 / 0.001 | 0.087 / 0.001 | 1.514: (1.404, 1.635) | < 0.0001 |
|  | *Length of shortest accessible path to nearest peak* | 0.823 / 0.022 | 1.482 / 0.029 | 0.539: (0.500, 0.579) | < 0.0001 |

**Supplementary file 1r**

**Supplementary file 1s**
